# Resistencia flexural de las resinas fluida convencional, fluida *bulk fill* y fluida de alta carga: estudio *in vitro*

**DOI:** 10.21142/2523-2754-1103-2023-161

**Published:** 2023-09-26

**Authors:** Edith Liliana Sulca Gonzales, Ana Isabel López-Flores

**Affiliations:** 1 División de Rehabilitación Oral, Carrera de Estomatología, Universidad Científica del Sur. Lima, Perú. 100069596@cientifica.edu.pe, alopezf@cientifica.edu.pe Universidad Científica del Sur División de Rehabilitación Oral Carrera de Estomatología Universidad Científica del Sur Lima Peru 100069596@cientifica.edu.pe alopezf@cientifica.edu.pe

**Keywords:** resinas compuestas, resistencia flexural, propiedades mecánicas, composite resins, flexural strength, mechanical properties

## Abstract

**Objetivo:**

Evaluar y comparar la resistencia flexural de las resinas fluida convencional, fluida bulk fill y fluida de alta carga.

**Materiales y métodos:**

Se confeccionaron treinta especímenes de 2 mm x 2 mm x 25 mm de las resinas fluidas Tetric N-Flow (TNF), Filtek Bulk Fill Flowable Restorative (FBF) y Beautifil Flow Plus F00 (BFP), las cuales se distribuyeron en tres grupos, de acuerdo con la marca (n = 10), y se almacenaron en agua destilada durante 24 horas a 37 °C. Se realizó la prueba de flexión de 3 puntos, de acuerdo con la norma ISO 4049, en una máquina de ensayo universal a una velocidad de 0,5 mm/min hasta la fractura. Los resultados se evaluaron con las pruebas de Anova y Tukey. P < 0,05.

**Resultados:**

Se encontró diferencias estadísticamente significativas entre los grupos con un valor de p = 0,011. Los grupos que mostraron diferencias estadísticamente significativas fueron los grupos BFP y TNF (p = 0,015) y los grupos BFP y FBF (p = 0,035), mientras que entre los grupos TNF y FBF no se encontró diferencia estadísticamente significativa.

**Conclusión:**

La resina fluida de alta carga presenta mejor resistencia flexural comparada con las resinas fluida convencional y fluida bulk fill, mientas que las resinas fluidas convencional y bulk fill no muestran diferencia estadísticamente significativa.

## Abstract (en)

**Objective:**

To evaluate and compare the flexural strength of conventional flowable, bulk fill flowable and highly filled flowable resins.

**Materials and methods:**

Thirty 2mm x 2mm x 25mm specimens of Tetric N-Flow (TNF), Filtek Bulk Fill Flowable Restorative (FBF) and Beautifil Flow Plus F00 (BFP) flowable resins were made up; they were distributed into three groups according to brand (n=10) and stored in distilled water for 24 hours at 37ºC. The 3-point bending test according to ISO 4049 was performed on a universal testing machine at a speed of 0.5 mm/min until fracture. The results were evaluated with Anova and Tukey tests. P<0.05.

**Results:**

Statistically significant differences were found between the groups with a value of p=0.011. According to the statistical analysis, the groups that showed statistically significant differences were the BFP and TNF groups (p=0.015) and the BFP and FBF groups (p=0.035), while no statistically significant difference was found in the TNF and FBF groups.

**Conclusion:**

The high-filled flowable resin presents better flexural strength compared to the conventional flowable and bulk fill flowable resins, while the conventional and bulk fill flowable resins have no statistically significant difference.

## INTRODUCCIÓN

Las resinas compuestas fluidas se desarrollaron en respuesta a la necesidad de un material con menos viscosidad que pueda adaptarse mejor a las paredes de una preparación ^1^^-^^4^, pues al aplicarse con jeringa se efectiviza su colocación en lugares poco accesibles. ^(1, 3, 4)^. Inicialmente, fueron indicadas para lesiones cervicales no cariosas, por ser un material que posee bajo módulo de elasticidad, esto les da un efecto de romper el estrés y así aliviar tensiones ^(2, 4)^. La desventaja de esta generación temprana de resinas fluidas es que, para lograr su fluidez, se tuvo que reducir la cantidad de relleno o incrementar los diluyentes, lo cual redujo sus propiedades mecánicas ^1^^-^^4^; esto ocasionó que tuvieran menor resistencia al desgaste que las resinas de alta viscosidad. En diversos estudios clínicos, se concluyó que tiene buena eficacia clínica cuando se aplicó en pequeñas cantidades, desde dos años, en restauraciones oclusales ^2^, hasta 5 años, en lesiones cervicales no cariosas ^3^. También se aplican en cavidades mínimas de clase I, II y III, selladores de fosas y fisuras, revestimientos de cavidades, en ortodoncia, entre otros ^1^.

Con el fin de disminuir el tiempo de trabajo, prevenir errores como la formación de burbujas y disminuir los tiempos clínicos, se desarrollaron las resinas tipo *bulk fill*^5^^-^^11^. Se realizaron modificaciones en la matriz y en el iniciador químico; además, se disminuyó el volumen de relleno inorgánico para reducir el estrés de contracción durante la fotopolimerización y así poder aplicarlas en incrementos de 4 mm ^(5, 8-10)^ hasta 5 mm ^(6, 7)^. Se aumentó la traslucidez para lograr el pase uniforme de la luz a niveles más profundos, a fin de efectivizar la polimerización ^(6, 12)^. Algunos materiales de baja viscosidad tienen una carga similar de relleno a la de los materiales de alta viscosidad, y propiedades mecánicas similares ^7^. Algunos estudios sugieren que la deflexión cuspal debido a la polimerización es menor cuando se utiliza resina fluida *bulk fill* en comparación con una resina fluida convencional, mediante la técnica incremental ^8^. También se han desarrollado resinas *bulk fill* que incorporan en su relleno ionómero de vidrio preactivado ^9^. Por su bajo contenido de relleno, la mayoría de estudios clínicos han sugerido el uso de las resinas *bulk fill* como base o revestimiento en restauraciones clase I y II, aunque también se ha demostrado su efectividad en restauraciones directas clase V por 1 año ^13^. En rehabilitación oral se pueden emplear para la reconstrucción en cavidades grandes antes de realizar una restauración parcial indirecta. Sin embargo, no estaría recomendado en restauraciones profundas clase II, por la baja biocompatibilidad con las células osteoblásticas ^7^.

Existe una correlación entre el porcentaje de relleno inorgánico en peso y volumen de las resinas compuestas fluidas y sus propiedades mecánicas ^14^. Por ello, se ha desarrollado una nueva generación de estos materiales en la que se incrementa el contenido de relleno ^14^^-^^19^. También influye el tamaño de relleno en la resistencia al desgaste de estos compuestos ^(15, 19)^. Estas resinas fluidas parecen ser más resistentes a la propagación de grietas, lo que incrementaría la resistencia al desgaste y les da el potencial de usarse en áreas de alto estrés ^(17, 19, 20)^. Por tanto, se podría indicar su uso también en restauraciones posteriores más grandes ^(15-17, 19)^ considerando que estos materiales presentan limitaciones en la profundidad de curado y la contracción de polimerización en incrementos de 4 mm ^16^. Algunos estudios determinaron que presentan propiedades comparables a las de las resinas compuestas e, incluso, tienen ciertas ventajas ^18^^,^^19^; sin embargo, no se podría asegurar el pronóstico a largo plazo, debido al efecto que causaría el impacto cíclico durante la masticación ^18^. La fuerza de unión es similar a la de las resinas compuestas en pasta ^21^. Se realizaron estudios clínicos para evaluar su rendimiento y se pudo observar que la resina fluida con alto relleno mostró una eficacia clínica comparable a la de una resina compuesta en pasta en restauraciones clase II por dos años ^22^, y en restauraciones posteriores durante 36 meses ^23^.

Estudios recientes evaluaron la resistencia flexural de distintas resinas fluidas al momento de fractura del espécimen, luego de ser sometido a la prueba de tres puntos en la máquina de pruebas universales. Haugen *et al*. ^7^ investigaron las propiedades físicas, químicas, mecánicas y biológicas de dos resinas fluidas *bulk fill* comparándolas con una resina convencional, y hallaron que la resistencia flexural de las resinas *bulk fill* fluidas fue mayor. Asimismo, Mirică *et al*. ^14^ evaluaron la correlación entre el porcentaje de relleno inorgánico en peso y en volumen de 11 compuestos de resina fluida y sus propiedades mecánicas; resultó que el porcentaje de relleno inorgánico estuvo muy bien correlacionado con las propiedades mecánicas de las resinas fluidas, principalmente el volumen para los valores de resistencia flexural. Por su parte, Tsujimoto *et al*. ^9^ evaluaron las propiedades de flexión de diferentes resinas fluidas inmediatamente después de la polimerización y luego de de 24 horas; y encontraron que las resinas fluidas de alta carga mostraron valores significativamente más altos en ambos momentos.

Las propiedades mecánicas de las resinas fluidas, entre ellas la resistencia flexural, han ido cambiando según la composición de estas. Actualmente, las propiedades mecánicas de las resinas fluidas han mejorado para expandir las opciones de tratamiento en una gama más amplia de situaciones clínicas. El uso de la técnica de resina inyectada es una opción clínica importante utilizada en rehabilitación oral para una temporalización o provisionalización larga. Por ello, la presente investigación evaluó y comparó la resistencia flexural de tres marcas de resina fluida con diferente composición química. La hipótesis nula por evaluar es que la resistencia flexural de las resinas fluida convencional, fluida *bulk fill* y fluida de alta carga es similar.

## MATERIALES Y MÉTODOS

El presente estudio fue evaluado por el Comité Institucional de Ética de la Universidad Científica de Sur y, por ser *in vitro* y no vulnerar los principios éticos, fue exonerado. Esta investigación utilizó como método la observación estructurada y el diseño de estudio fue experimental *in vitro*. Se confeccionaron treinta especímenes de las resinas fluidas Tetric N-Flow (TNF), Filtek Bulk Fill Flowable Restorative (FBF) y Beautifil Flow Plus F00 (BFP), los cuales se distribuyeron en tres grupos, de acuerdo con su marca (n = 10) (Tabla 1).

**Tabla 1:** Resinas fluidas utilizadas en este estudio

Material	Composición química	Color	Fabricante	Lote
Tetric N-Flow	Bis-GMA, UDMA, TEGDMA/vidrio de bario, trifluoruro de iterbio, sílice altamente dispersado y óxido mezclado, iniciadores, estabilizadores y pigmentos. Relleno 63% peso y 39% volumen	A1	Ivoclar Vivadent AG Schaan/Liechtenstein	Z03RD9
Filtek Bulk Fill Flowable Restorative	Bis-GMA, Bis-EMA, UDMA/zirconia, silica, trifluoruro de iterbio. Relleno 64,5% peso y 42,5% volumen	A1	3M ESPE (St. Paul, MN, EE. UU.)	NE31699
Beautifil Flow Plus F00	Bis-GMA, TEGDMA, relleno S-PRG basado en fluoroboroaluminosilicato. Relleno 67,3% peso y 47% volumen	A1	Shofu Inc (Kioto, Japón)	122191

Bis-GMA: Bisfenol-A glicol metacrilato

UDMA: Dimetacrilato de uretano

TEGDMA: Dimetacrilato trietilenglicol

Bis-EMA: Bisfenol-A hexaetoxilado dimetacrilato

S-PRG: Superficie prerreactivada con ionómero de vidrio

El número final de muestras se obtuvo luego del estudio piloto aplicando la fórmula de tamaño muestral para comparar dos medias. 

Los criterios de inclusión considerados fueron barras de resina fluida fotoactivadas con una fuente de luz LED, especímenes de resina fluida con medidas de 25 mm x 2 mm x 2 mm, de acuerdo con los estándares universales de la ISO 4049; especímenes de resina fluida debidamente pulidos, barras de resinas fluidas de color A1. Se excluyeron los especímenes que no cumplan con los criterios de inclusión. 

**Figura 1:**
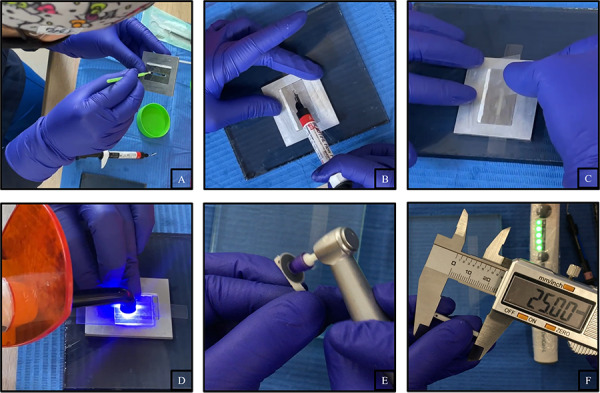
Confección de los especímenes. A. Colocación de vaselina con un microbrush en la matriz. B. Aplicación de resina fluida sobre una platina de vidrio con tiras de poliéster. C. Colocación de una tira de poliéster sobre la resina fluida. D. Fotoactivación con una lámpara de luz led presionando los portaobjetos de vidrio. E. Pulido de excesos de resina fluida. F. Verificación de las medidas con un calibrador digital.

Los especímenes se confeccionaron en una matriz proporcionada por el laboratorio con dimensiones internas de 2 mm x 2 mm x 25 mm. Se aisló este molde aplicando vaselina con microbrush, luego se aplicó cada tipo de resina fluida presionándola entre dos portaobjetos de vidrio cubiertos con tiras de poliéster sobre una platina de vidrio. Se fotoactivó con una lámpara de luz led de la marca Elipar Deep Cure 3M ESPE, con una potencia de 1470mw/cm^2^, por la parte superior e inferior en tres etapas de 40 segundos a lo largo de las muestras; luego, estas se retiraron cuidadosamente del molde tras la polimerización y el material de resina fluida residual se eliminó de ambas superficies puliéndolas con discos de pulido. Se examinó visualmente con una lupa que no exista presencia de burbujas o algún defecto en los especímenes; posteriormente, sus dimensiones definitivas se verificaron con un calibrador digital. Finalmente, las muestras se almacenaron en agua destilada durante 24 horas a 37 °C antes de realizar las pruebas ^5^^,^^24^ (Fig. 1).

**Figura 2:**
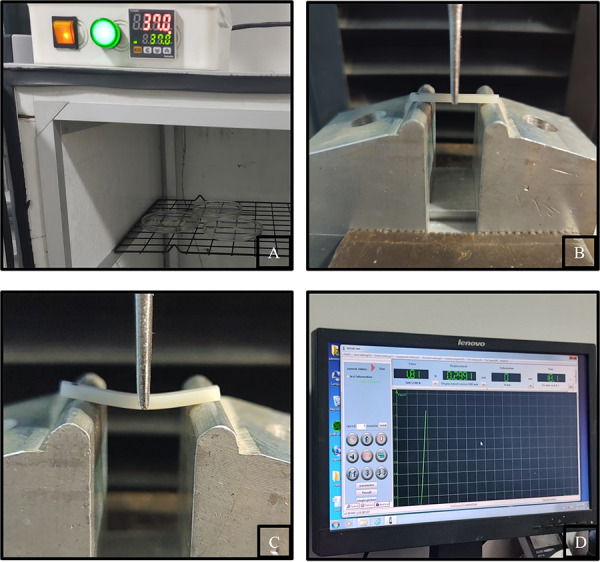
Ensayo mecánico. A. Almacenamiento de los especímenes. B. Las muestras se colocaron sobre soportes con un espacio de 20 mm entre ellos. C. Flexión del espécimen previo a la fractura. D. Se calcularon los valores de resistencia flexural.

La prueba de resistencia flexural se realizó utilizando la prueba de flexión de 3 puntos, de acuerdo con la norma ISO 4049, en una máquina de ensayo universal (LG, Modelo: CMT-5L, Serie: 7419, Corea) en High Technology Laboratory Certificate. Las muestras se colocaron sobre soportes con un espacio de 20 mm entre ellos y se realizó la prueba a una velocidad de 0,5 mm/min hasta la fractura. Los valores de resistencia flexural (MPa) se calcularon utilizando la siguiente fórmula: FS = 3Fmax1/2bh^2^, donde FS es la resistencia flexural, Fmax es la carga aplicada (N), l es el espacio entre los soportes (20 mm), b es el ancho (2 mm) y h es el espesor (2 mm) (Fig. 2). Todos los datos obtenidos fueron llenados en una ficha de recolección de datos.

El análisis estadístico se realizó con el programa SPSS versión 25; se analizó la estadística descriptiva a través de las medidas de tendencia central y dispersión. La distribución de datos se analizó utilizando la prueba de Shapiro-Wilk. La prueba de significancia estadística con Anova de un factor y comparaciones múltiples de Tukey P < 0,05. 

## RESULTADOS

La resistencia flexural encontrada en el grupo TNF fue de 96,73 ± 6,09 MPa; en el grupo FBF fue de 97,99 ± 8,34 MPa, y en el grupo BFP fue de 107 ± 2,60 MPa. Cuando se comparó la significancia entre los 3 grupos, se halló diferencias estadísticamente significativas con la prueba Anova de un factor con un valor de p = 0,011). Con la prueba de comparaciones múltiples de Tukey, BFP y TNF mostraron diferencias estadísticamente significativas (p = 0,015), al igual que los grupos BFP y FBF (p = 0,035); entre los grupos TNF y FBF no se encontró diferencia estadísticamente significativa (Tabla 2).

**Tabla 2:** Evaluación de la resistencia flexural en las resinas fluida convencional, fluida *bulk fill* y fluida de alta carga

Grupo	Resistencia flexural (MPa)	Valor de significancia (p)
1. TNF	96,73 ± 6,09A	0,927 (entre grupo 1 y 2)
2. FBF	97,99 ± 8,34A	0,035 (entre grupo 2 y 3)
3. BFP	107 ± 2,60B	0,015 (entre grupo 1 y 3)

Letras diferentes indican diferencia estadísticamente significativa.

TNF: Tetric N-Flow

FBF: Filtek Bulk Fill Flowable Restorative

BFP: Beautifil Flow Plus F00

## DISCUSIÓN

El propósito de esta investigación fue evaluar la resistencia flexural de tres resinas fluidas con distinta composición química. La premisa planteada fue que la resistencia flexural de las resinas fluidas convencionales, *bulk fill* y de alta carga difiere. Para la prueba de resistencia flexural se elaboraron 10 muestras de cada tipo de resina fluida y se llevó a cabo la evaluación de flexión de 3 puntos utilizando una máquina de ensayo universal. 

Los resultados de este estudio evidenciaron la presencia de diferencias estadísticamente significativas entre los grupos, con un valor de p = 0,011. Según el análisis estadístico, los grupos que mostraron estas diferencias fueron BFP y TNF (p = 0,015) y los grupos BFP y FBF (p = 0,035), mientras que entre los grupos TNF y FBF no se encontró diferencia estadísticamente significativa. Estos resultados podrían deberse a que la resina fluida de alta carga (BFP) tiene mayor porcentaje de relleno inorgánico (67,3% peso y 47% volumen) y a que esto le brinde una resistencia flexural superior en contraste con las demás resinas fluidas. El porcentaje de relleno inorgánico presente en la resina fluida *bulk fill* (FBF) (64,5% peso y 42,5% volumen) es ligeramente mayor al porcentaje de relleno inorgánico de la resina convencional (TNF) (63% peso y 39% volumen), esto podría explicar por qué la resistencia flexural de FBF fue mayor a la de TNF; no obstante, esta disparidad no alcanzó significancia estadística.

Diversos estudios han comparado la resistencia flexural de las resinas compuestas. Haugen *et al*. ^7^ llevaron a cabo una investigación sobre las propiedades físicas, químicas, mecánicas y biológicas de dos resinas fluidas *bulk fill*, una de ellas evaluada en el presente estudio (FBF), y las comparó con una resina viscosa. Encontró que las resinas fluidas *bulk fill* presentaron una mayor resistencia flexural. En el presente estudio, si bien es cierto que se compararon resinas de una misma viscosidad, no se encontraron diferencias estadísticamente significativas en cuanto a la resistencia flexural entre FBF y la resina fluida convencional Tetric N-Flow (TNF).

De igual modo, los resultados corroboran lo encontrado en el estudio de Mirică *et al*. ^14^, quienes reportaron que el porcentaje de relleno inorgánico registrado estaba muy bien correlacionado con las propiedades mecánicas de las resinas fluidas. A pesar de las diferencias en promedio encontradas, los resultados estadísticos son similares a los hallazgos encontrados en el presente estudio, donde se expresa que la resina fluida de alta carga siempre tiene un mejor comportamiento en cuanto a resistencia flexural. 

En contraste, Tsujimoto *et al*. ^9^, al evaluar la resistencia flexural de resinas fluidas de alta carga después de 24 h, encontraron que esta propiedad fue significativamente superior para BFP comparada con la del material compuesto convencional. Asimismo, la mayoría de los grupos de resina fluida fueron similares a las del material compuesto *bulk-ﬁll* (FBF), mientras que, en el presente estudio, la resistencia ﬂexural del mismo compuesto *bulk-ﬁll* sí mostró diferencias significativas cuando fue comparado con una resina fluida de alta carga.

El análisis de esta propiedad en los distintos tipos de resinas fluidas ayudará al profesional clínico a tomar decisiones más acertadas al seleccionar el material de restauración adecuado para situaciones clínicas específicas, como temporizaciones prolongadas o restauraciones de clase V, por ejemplo. En ese sentido, sería importante realizar nuevas investigaciones sobre estos materiales, simulando el envejecimiento clínico mediante el termociclado, y evaluar sus propiedades mecánicas.

## CONCLUSIÓN

La resina fluida de alta carga presenta mejor resistencia flexural comparada con las resinas fluida convencional y fluida *bulk fill*, mientras que las resinas fluidas convencional y *bulk fill* no tienen diferencia estadísticamente significativa.

## Fuente de financiamiento:

La presente investigación fue autofinanciada
